# Patterned Drug-Eluting Coatings for Tracheal Stents Based on PLA, PLGA, and PCL for the Granulation Formation Reduction: In Vivo Studies

**DOI:** 10.3390/pharmaceutics13091437

**Published:** 2021-09-09

**Authors:** Olga A. Sindeeva, Ekaterina S. Prikhozhdenko, Igor Schurov, Nikolay Sedykh, Sergey Goriainov, Arfenya Karamyan, Ekaterina A. Mordovina, Olga A. Inozemtseva, Valeriya Kudryavtseva, Leonid E. Shchesnyak, Rimma A. Abramovich, Sergey Mikhajlov, Gleb B. Sukhorukov

**Affiliations:** 1 Skolkovo Innovation Center, Skolkovo Institute of Science and Technology, 3 Nobel Str., 143005 Moscow, Russia; 2 Science Medical Center, Saratov State University, 83 Astrakhanskaya Str., 410012 Saratov, Russia; prikhozhdenkoes@gmail.com (E.S.P.); mordovina_ekaterina@mail.ru (E.A.M.); inozemtsevaoa@mail.ru (O.A.I); 3 Innovative Engineering Technologies Institute, Peoples Friendship University of Russia (RUDN University), 6 Mikluho-Maklaya Str., 117198 Moscow, Russia; schurov.ig@yandex.ru (I.S.); svarog988@mail.ru (N.S.); goryainovs@list.ru (S.G.); karamyan-as@rudn.ru (A.K.); shchesnyak-le@rudn.ru (L.E.S.); abr-rimma@yandex.ru (R.A.A.); newshatel@mail.ru (S.M.); 4 Nanoforce Ltd., School of Engineering and Materials Science, Queen Mary University of London, Mile End Road, London E1 4NS, UK; v.kudriavtceva@qmul.ac.uk

**Keywords:** tracheal stents, drug-eluting coatings, microchamber array, PLA, PCL, PLGA, methylprednisolone sodium succinate, in vivo study, Soviet chinchilla rabbits

## Abstract

Expandable metallic stent placement is often the only way to treat airway obstructions. Such treatment with an uncoated stent causes granulation proliferation and subsequent restenosis, resulting in the procedure’s adverse complications. Systemic administration of steroids drugs in high dosages slows down granulation tissue overgrowth but leads to long-term side effects. Drug-eluting coatings have been used widely in cardiology for many years to suppress local granulation and reduce the organism’s systemic load. Still, so far, there are no available analogs for the trachea. Here, we demonstrate that PLA-, PCL- and PLGA-based films with arrays of microchambers to accommodate therapeutic substances can be used as a drug-eluting coating through securely fixing on the surface of an expandable nitinol stent. PCL and PLA were most resistant to mechanical damage associated with packing in delivery devices and making it possible to keep high-molecular-weight cargo. Low-molecular-weight methylprednisolone sodium succinate is poorly retained in PCL- and PLGA-based microchambers after immersion in deionized water (only 9.5% and 15.7% are left, respectively). In comparison, PLA-based microchambers retain 96.3% after the same procedure. In vivo studies on rabbits have shown that effective granulation tissue suppression is achieved when PLA and PLGA are used for coatings. PLGA-based microchamber coating almost completely degrades in 10 days in the trachea, while PLA-based microchamber films partially preserve their structure. The PCL-based film coating is most stable over time, which probably causes blocking the outflow of fluid from the tracheal mucosa and the aggravation of the inflammatory process against the background of low drug concentration. Combination and variability of polymers in the fabrication of films with microchambers to retain therapeutic compounds are suggested as a novel type of drug-eluting coating.

## 1. Introduction

Stent placement is a standard and often the only way for treating airway obstructions (for instance, malacia, fistula [1], and oncology) in clinical and veterinary practice [2]. Mainly, there are two types of stents used: covered (silicone, metallic, hybrid stents—metallic with silicone coating) and uncovered (expandable metallic) stents [3,4,5]. Covered stents block proliferation granulation or tumor tissue through the stent, but the cover hampers expectoration of sputum. The accumulation of sputum between the stent and the trachea is a favorable environment of respiratory infection increase [6] and often accompanied by pneumonia, up to the removal of the lung or death [6]. Uncovered expandable metallic stents do not contribute to such pronounced accumulation of sputum; however, they allow granulation tissue to proliferate within the stent, contribute to restenosis up to complete blockage of the stent lumen [2,7].

The main reason why granulation tissue develops impairs the microcirculation associated with mechanical exposure (friction and contact pressure) on the airway wall by the stent [8,9]. The micromovements of the stent damage the tracheal mucosa and lead to edema and inflammation, which contribute to the formation of granulation tissue. Stent removal due to granulation tissue hyperplasia is challenging [10].

Systemic administration of steroid drugs in high dosages slows down granulation tissue overgrowth [11] due to their pronounced anti-inflammatory effects, but in turn, leads to other long-term side effects [12]. A revolutionized systemic side effects reduction method is a drug-eluting stent that can deliver the drug directly to the diseased area [13,14,15,16]. Biodegradable materials [17] and 3-D printing [18] have high potential in this area, but medical-grade materials are in the development stage [19]. Although, the PCL-based biodegradable stent exhibited mechanical strength, steady release of cisplatin for more than four weeks in vitro and sustained cisplatin levels in rabbit trachea for more than five weeks with a minimum drug level in blood [20]. Several promising cases of using the bioresorbable stents in clinical practice have already been noted [21].

Another promising approach is metallic stents with a drug-eluting coating used in cardiology for many years but there are no commercially available analogs for the trachea. Today, several composite coating options can be used as candidates [22,23,24,25,26]. The electrospun PCL-based coatings for metal stents have excellent mechanical characteristics as reported in [27]. PLA-based nanofiber membranes with silver nanoparticles were used to cover a self-expandable metallic stent and showed antibacterial activity, cytocompatibility and suppressed tracheal stenosis in rabbit by reducing inflammation and collagen deposition [28]. At the same time, the composite materials where the drug and polymer are blended have an uncontrolled release profile that cannot be changed. The development of complications after stent implantation occurs individually in all patients [1], which requires a customizable and personalized approach to therapy. In addition, drug choices are generally limited to poorly water-soluble compounds.

Here, we report on a new prospective drug-eluting coating based on polymer films with microchamber arrays [29,30]. The shell of such coatings can be synthesized by the adsorption of hydrophilic oppositely charged polyelectrolytes (layer-by-layer method) [31,32,33], as well as from hydrophobic polymers: PLA [33], PLGA [34], and PCL [35]. Microchamber arrays are suitable for loading a wide range of both poorly and well-water-soluble substances with low [34,36] and high [37,38] molecular weight. Such coatings have been demonstrated to be sensitive to ultrasound [33,34] and NIR-laser radiation [39] after shells were modified with photo adsorbent agents such as gold coating [40,41] or nanoparticles [37] and carbon dots aggregates [38,42]. The site-specific laser-induced release of glutamate hydrochloride stimulated the response from neuronal-type cells (N2A) located near the open chambers [36], controlled nerve growth factor release stimulated the growth and proliferation [37]. It was shown in vivo that ultrasound-indicated adrenaline release from PLGA-based microchamber arrays led to the immediate vascular reaction [34]. The sensitivity of microchamber arrays to external stimuli makes it possible to release the drug on demand and individually select the therapy regimen. It has already been shown in in vitro studies that such coatings based on PLA can be used to functionalize the surface of coronary stents [43]. Here, we show that films with microchamber arrays based on various biocompatible polymers (PLA, PLGA, and PCL) can be used for methylprednisolone sodium succinate loading and fixed on the surface of an expandable nitinol stent. In this study, we look at how the type of polymer affects the coating’s mechanical stability and the therapeutic effect.

## 2. Materials and Methods

### 2.1. Materials

Poly (D,L-lactide-co-glycolide) (PLGA)—lactide:glycolide (50:50), Mw ∼30,000–60,000; polycaprolactone (PCL)—Mw ∼80,000; polylactic acid (PLA)—Mw ∼60,000; fluorescein isothiocyanate-dextran (FITC-dextran) Mw ∼5000, and chloroform were obtained from Sigma-Aldrich, Germany. Anti-inflammatory drug—methylprednisolone sodium succinate (Solu-Medrol) was obtained from Pfizer, Belgium. The Poly (dimethylsiloxane) (PDMS) kit (Sylgard 184) was purchased from Dow-Corning, Midland, USA. DexStent-TN (nitinol tracheal stents, d = 8 mm, l = 60 mm; and delivery systems, d = 10 Fr, l = 470 mm) were obtained from Dextronix, France.

### 2.2. Fabrication of Sealed Free-Standing Polymer Films with Microchambers Array Containing Methylprednisolone Sodium Succinate and Fluorescein Isothiocyanate-Dextran

**Figure 1:**
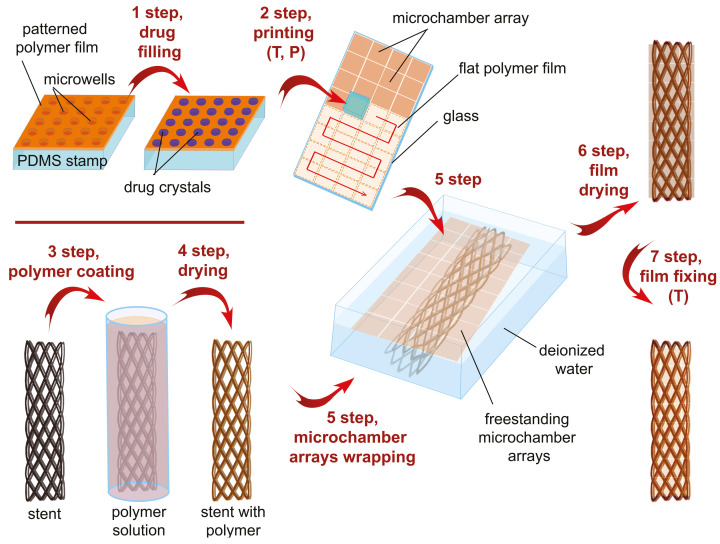
General scheme of tracheal stent surface functionalization by biodegradable microchamber arrays containing anti-inflammatory drug.

For tracheal stent coating, microchamber array film was used, consisting of a patterned film with microwells, a drug, and a flat film (Figure 1). The patterned film was formed using a prefabricated PDMS stamp with wells [44,45] (stamp size, 8 × 8 mm; microwells diameter, 10 μm; depth, 5 μm; center-to-center distance, 20 μm). The stamp was dipped for 3 s in PCL (1 wt%), PLA (1 wt%), or PLGA (1.5 wt%) chloroform solutions and dried. As a result, the thin-patterned polymer film was formed on the PDMS stamp.

In the first step, 1 μL of methylprednisolone solution (20 mg/mL) was applied to the surface of a patterned film and slowly dried. The drug solution was continuously dispensed over the surface of the patterned film using a pipette tip to ensure that the drug was evenly filled into the microwells. Drug crystals remained only inside the wells after the solution dried due to the hydrophobic properties of the chosen polymers [46].

In the second step, the microscope slides (76 × 26 mm) were dipped in similar polymer solutions and dried to form thin flat films. Then, the patterned film located on the PDMS stamp surface was printed onto the flat film. For printing, a microscope slide with the flat film was placed on a heated flat surface of a magnetic stirrer (70 °C), and a pressure of 2 kg/cm${}^{2}$ was applied to the PDMS (20 s). After printing a tiny patterned film, the stamp was removed, and the received microchamber array was 8 × 8 mm. Then, a new film was synthesized on the surface of the PDMS stamp and filled with the drug. The next patterned film was printed near the previous one on the same flat film. The procedure was repeated 12 times on one microscope slide (4 × 3 microchamber arrays, 32 × 24 mm of coating). Coating from three slides was used to cover the tracheal stent completely.

### 2.3. Functionalization of Tracheal Stent Surface by Microchamber Array Film

We immersed the nitinol stent in a 1.5 wt% PLGA solution for uniform coating (3rd step, Figure 1); then, the stent was dried in a jet of compressed air to remove excess polymer and avoid film formation between the wires. To detach the hydrophobic film of the microchamber array from the glass surface, we immersed the glass with the film in deionized water for 2 minutes. As a result, the straightened film with microchamber arrays floated on the water surface. We then submerged the PLGA-coated stent under the floating film and lifted it. As a result, the film evenly covered the stent with little overlap. Then, we gently dried the coated stent for 30 min at 37 °C. At the last—seventh step, the coated stent was placed in a sterile centrifuge tube (15 mL) and heated for 15 minutes at 55 °C. Chloroform evaporates more actively from polymers with increasing temperature, the viscosity of the polymer increases with temperature, and heat shrinkage helps the film adhere more tightly to the PLGA-coated nitinol. The proposed method makes it possible to securely and evenly fix the film on the nitinol stent, ensuring the load distribution on the polymer coating over the entire stent area.

### 2.4. Characterization Technique

Scanning Electron Microscopy (SEM) measurements were performed with a VEGAIII (TESCAN, Czech Republic) microscope at an operating voltage of 30 kV. Before measurement, gold was sprayed onto the sample (∼5 nm gold layer) using an Emitech K350 sputter-coater (Quorum Technologies Ltd, Ashford, UK).

Confocal laser scanning microscope (Leica TCS SP8 X) equipped with white light laser (470–670 nm excitation range with 1 nm precision) was used for fluorescent cargo estimation from PCL, PLA, and PLGA coating of tracheal stents after deformation in a delivery system provided by the manufacturer with DexStent-TN nitinol stents. By deformation, we mean the mechanical effect exerted on the stent when it is packed into the delivery system (precisely, decrease in diameter, increase in length). FITC-dextran (concentration, 10 mg/mL) was encapsulated as cargo and excited at 495 nm with a 505–540 nm detection range. Z-scans with a step of 1 μm were collected for 3D visualization of microchamber array films.

Gas chromatography-mass spectrometry (GC-MS) analysis of samples was performed using Saturn 2100T (Varian Inc., Middelburg, The Netherlands) equipped with gas chromatograph Varian 3900 (Varian Inc., Middelburg, The Netherlands). This method has been used to estimate the amount of methylprednisolone sodium succinate encapsulated in the coatings as one of the standard methods for the quantitative determination of steroidal substances [47]. GC separation conditions: capillary column VF-5MS (Agilent Technologies, Santa Clara, CA, USA; length, 30 m; internal diameter, 0.25 mm; phase thickness, 0.25 μm); the carrier gas was helium; the velocity of the carrier gas was 1.5 mL/min; the injector temperature was 280 °C; the initial temperature of the chromatograph furnace was 200 °C, then there was isotherm for 1 min; after that, it was heated at the speed of 10 °C/min up to 290 °C, held up for 10 min. The total analysis time was 20 min. Prior to mass-spectrometry analysis, 1 mL of chloroform:methanol mixture in 80:20 ratio was added to all samples. Each sample was analyzed three times. Mass spectra registration mode: ionization energy 70 eV, source temperature 270 °C, scanning in the range 40–600 Da. The volume of the injected sample is 1 μL. The NIST 14 mass spectral database was used to identify the compounds.

AlamarBlue Cell Viability Reagent based on resazurin was used to function as an L929 cell health indicator (to measure viability quantitatively) 24 h after growing on the coating’s surface. Upon entering living cells, resazurin is reduced to resorufin, a compound that is red in color and highly fluorescent.

### 2.5. In Vivo Study

Adult male Soviet chinchilla rabbits were randomly assigned for 3 groups: intact animals; control—a bare nitinol stent; experimental—nitinol stent with 3 types of drug-eluting coating. The stents were deployed endoscopically under general anesthesia and fluoroscopic guidance.

All animals were observed for any sign of respiratory distress or deterioration in general health during the 10-day duration of this experiment. Then, the rabbits were euthanized on day 10 by anesthesia overdose. The following pathomorphological aspects were evaluated: tracheal stenosis, granulation tissue, inflammation, and secretions in the tracheal lumen.

Animal care protocols complied with the European Communities Council Directive (86/609/EEC), with prior approval from our local Ethics Committee of Peoples’ Friendship University (protocol no. 1 from 9 August 2019).

## 3. Results and Discussion

### 3.1. Functionalization of Tracheal Stent Surface by PLA, PLGA, and PCL Coatings Based on Microchamber Arrays

**Figure 2:**
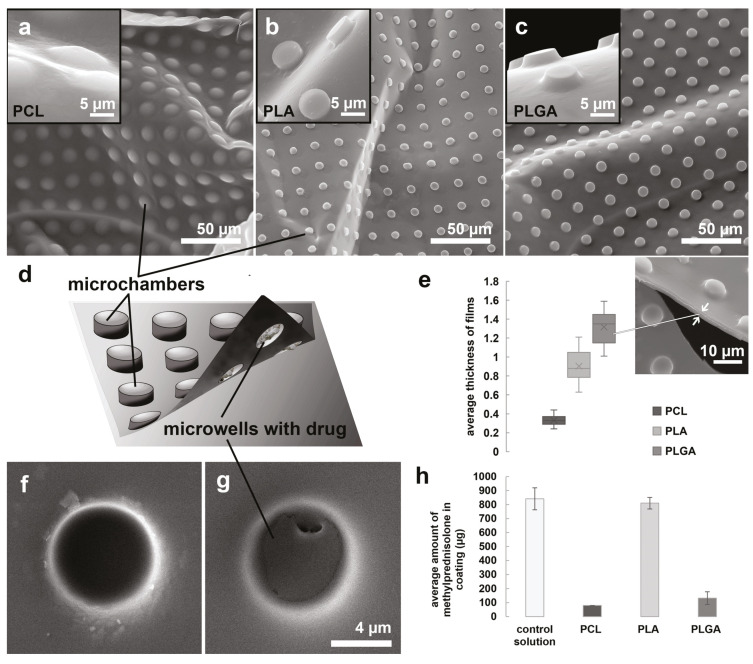
SEM images of the outer surface of microchamber arrays based on PCL (**a**), PLA (**b**), and PLGA (**c**). General scheme of microchamber array structure (**d**). The average thickness of microchamber array films based on PCL, PLA, PLGA (**e**). The inner surface of the empty (**f**) and filled with methylprednisolone sodium succinate (**g**) microwells. The average amount of methylprednisolone measured with GC-MS in the stent coating based on PCL, PLA, and PLGA (**h**).

The shape of microchambers differed significantly depending on the selected type of polymer when using the same PDMS stamp (Figure 2a–c). The possible explanation could be caused by the different mechanical properties of these three polymers and how the polymer solution wets the surface of the PDMS stamp and hence the variable polymer layer thickness. The most remarkable difference was manifested in PCL-based microchambers. For this polymer, the outer surface of the microchambers looked like hemispheres, while for other polymers (PLGA and PLA), it generally repeated the shape of the PDMS stamp microwells (cylinders). At the same time, the type of polymer selected did not significantly affect the behavior of the encapsulated solution on the surface of the patterned film, but different shapes of chambers might result in loading capacity. As a result, large precipitates of the methylprednisolone sodium succinate were formed inside the microwell after drying the solution (Figure 2d–g). We have chosen the methylprednisolone sodium succinate as a standard steroid drug for the reason that it is often used to reduce inflammation and prevent tracheal restenosis after surgery (including stenting) [48,49,50,51].

Note that for methylprednisolone sodium succinate, we used the encapsulation method from a small volume (1 μL) of a solution with a high concentration—20 mg/mL of the substance. The solution was dried on a hydrophobic microrelief surface with a uniform droplet distribution with a micropipette tip. This approach differed from the previously described methods for encapsulating hydrophilic molecules in microchambers based on hydrophobic polymers [33,39,40,42,52].

The methylprednisolone sodium succinate solution strongly wets the hydrophobic surface at high concentrations (more than 100 μg). We could not encapsulate a significant amount of this drug by separating tiny droplets held by capillary forces in the microrelief during removal of a large droplet of concentrated solution (method based on Wenzel’s state [46,53]). We have successfully used and described this method earlier in our previous works with epinephrine hydrochloride [34] and glutamate hydrochloride [36]. At the same time, the solution poorly wets the polymer surface; therefore, the drop cannot be uniformly distributed over the surface for free drying, as in the case of the nerve growth factor solution [37].

Note that we used the minimum allowable concentration of polymers forming a film on the PDMS surface. So, for PLA [54] and PCL [35], 1 wt% was enough, while for PLGA the concentration was higher—1.5 wt% [34]. The thickness of the resulting films with microchamber arrays differed significantly between the PLA and PCL and was maximum for PLGA (Figure 2e).

Immersion of films with microcontainer arrays in deionized water was accompanied by rapid drug release from PCL- and PLGA-based films. After the procedure, they contain only 9.5% and 15.7%, respectively. The PLA-based films were hermetic and held the cargo during this manipulation. The amount of substance remaining in the film measured with gas chromatography-mass spectrometry was comparable to the amount of this substance in the same volume of the solution used for capsulation and 96.3% (Figure 2h). Note that immersing the films in water for 2 minutes is one step of the stent coating (Figure 1, 5th step).

The choice of an adequate model for in vitro estimation of release from coatings is rather tricky in the case of an endotracheal implant. This is primarily due to the significant contribution of bacteria (living on the tracheal mucosa) to the degradation of the microchamber shell [55] and the drug release from them, respectively. Our early studies showed principal differences in the rate and character of the polymer shell degradation in vitro and in vivo (even with subcutaneous implantation) [34]. Therefore, in this study, we did not show the release profile of methylprednisolone sodium succinate in vitro and focused exclusively on evaluating the therapeutic effect of coatings.

### 3.2. Comparison of PLA, PLGA, and PCL Coatings Mechanical Properties

**Figure 3:**
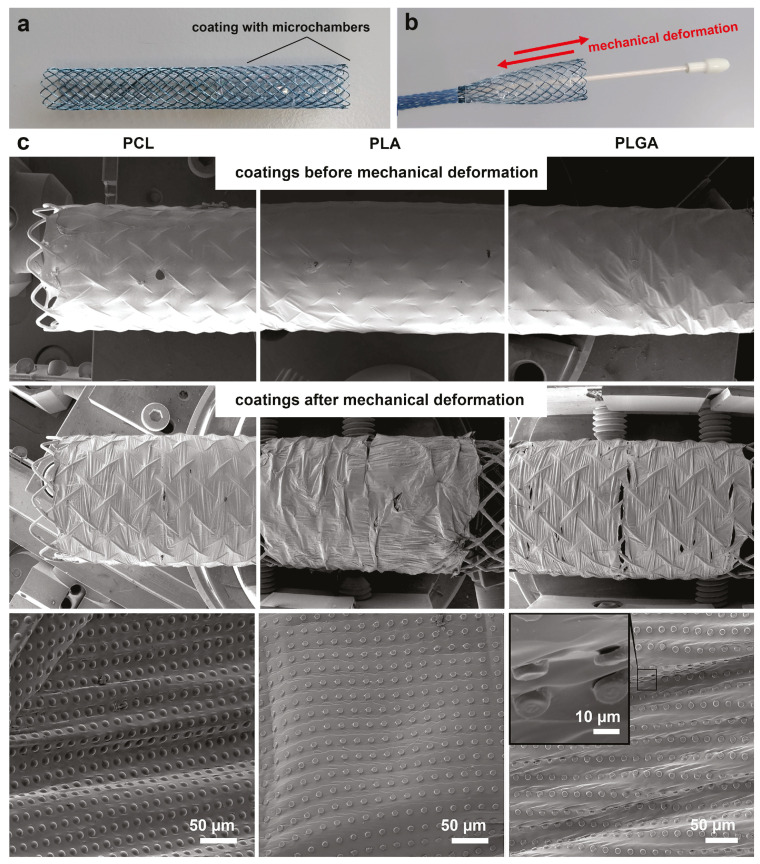
Typical photo of the stent with a coating based on microchamber arrays before (**a**) and after deformation in a delivery system (**b**). Stent size, 60 × 8 mm. SEM images of the stent with microchamber arrays based on PCL, PLA, and PLGA before and after deformation in a delivery system (**c**).

During implantation, the coated tracheal stent (Figure 3a) should be folded in the delivery device. The physician opens the stent when the delivery device is correctly positioned in the trachea (Figure 3b). The Nitinol Shape Memory Alloy allows the stent to be restored to its original state when exposed to body temperature [3]. In this case, the most crucial requirement for the stent coating is sufficient mechanical resistance to packaging and subsequent opening in the delivery device.

The packaging of the selected type of stent in the delivery device was accompanied by a significant decrease in diameter (from 8 mm to 3 mm) and an increase in length (from 60 mm to 90 mm). Fixation of the coating with microchamber arrays on nitinol due to the temperature effect and the presence of PLGA on it allows the stent to be easily closed and opened in the delivery device (Figure 3b,c). After mechanical deformation, the film remains uniformly stretched on the nitinol surface (which acts as “an umbrella”). The packaging of the coated stent without PLGA layer led to the separation between film and stent (Figure S1).

The images obtained using SEM showed that the packaging of the coated stent in the delivery device leads to a stretching of the polymer film between the chambers. We found only pinpoint damage to individual PLGA-based chambers (Figure 3d). Microchambers looked non-damaged for other polymers.

**Figure 4:**
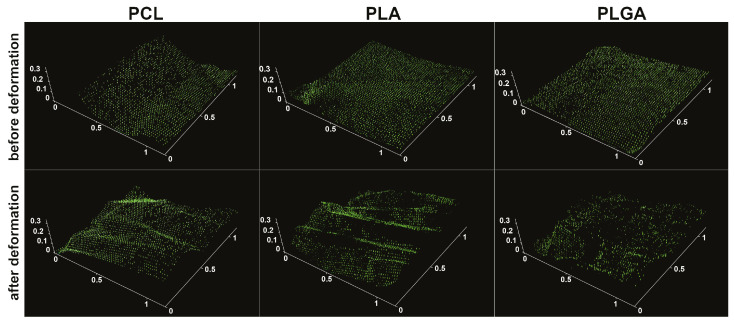
Confocal images of stents with PCL, PLA, and PLGA coating based on microchamber arrays before and after deformation in a delivery system. Cargo is FITC-dextran (5 kDa). The scale is in mm.

FITC-dextran (5 kDa) was encapsulated into the microchambers for a more detailed analysis of the mechanical stability of coatings (Figure 4). After mechanical deformation, the stent was placed for 15 min in deionized water to release the fluorescent cargo from the damaged chambers. Confocal microscopy showed that the high-molecular-weight cargo was in most PCL- and PLA-based microchambers after mechanical deformation, indicating their tightness. The damage to the PLGA-based microchambers was more significant than PCL- and PLA-based ones, consistent with the SEM data (Figure 3d).

### 3.3. In Vivo Studies of the Use of PLA, PLGA, and PCL Coatings to Reduce Granulation Tissue

Preliminary biocompatibility studies of the PCL-, PLA-, and PLGA-based coatings for tracheal stents did not reveal a cytotoxic effect on L929 murine fibroblast cells (Figure S2a). L929 cells grown on the coating’s surface had normal morphology (Figure S2b). Other authors also reported biocompatible and not toxic films obtained from a PLA solution in chloroform for cells culturing: preosteoblastic MC3T3-E1 cells [56] and adipose-derived stem cells [57].

**Figure 5:**
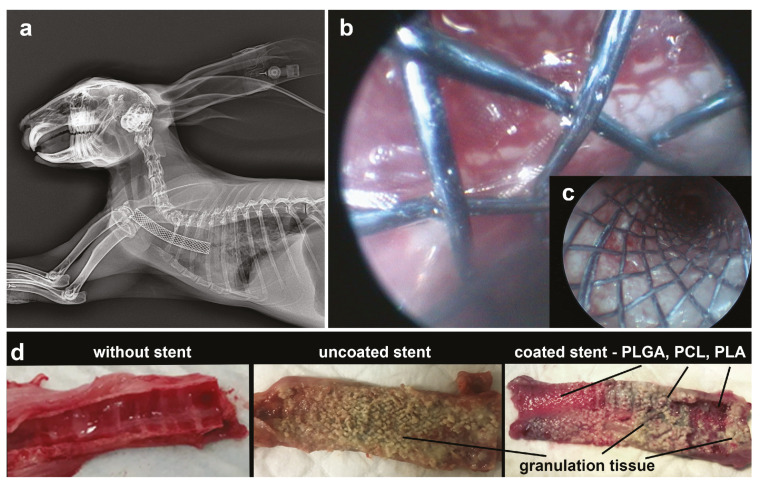
X-ray (**a**) and endoscopic images (**b**,**c**) during stent implantation surgery. On endoscopic images, the film with microchamber arrays is visible (**b**). Photo of the normal trachea and trachea 10 days after stent implantation with and without coating (**d**).

Tracheal stents were implanted in anesthetized rabbits under X-ray and endoscope control (Figure 5a,b). Uncoated stents were implanted in the control group of animals. Stents with three types of films with microchambers arrays (PLGA, PCL, PLA simultaneously) were implanted in the experimental group. The intact group included animals without stents. Figure 5b,c clearly shows the patterned structure of films with microchamber arrays on a stent surface installed in the trachea. After 10 days, the animals were killed by overdose. The therapeutic effect was assessed according to the results of the pathological analysis of the trachea structure. In the intact group, the tracheal mucosa had a normal pink color, while in the control group, there was a strong development of granulation tissue along the entire stent length (Figure 5d). In the experimental group, the trachea looked different depending on the type of coating in contact with it. The boundary between different polymer coatings was distinguishable. The greatest growth of granulation tissue was observed in the area where the PCL-based coating was located. The morphological state of the trachea here was comparable to the state of the trachea in the control group. The granulation tissue was much less pronounced or completely absent in the area which contacted the PLA-based coating. The best therapeutic result was achieved in the area which contacted the PLGA-based coating. Here, granulation tissue was absent, despite clear signs of inflammation. The highest incidence of stenosis was observed with the control group. This evidence is consistent with a variety of clinical data [58,59]. Less stenosis was recorded for the experimental group in the area with PLGA-coating.

In clinical practice, intravenous administration of methylprednisolone sodium succinate in dosage 2 × 3 mg/kg for 5 days (10 doses) is used to prevent the development of granulation tissue after tracheal surgery [51]. This dosage is applied systemically and considered to be effective and safe. In our study, we applied the methylprednisolone sodium succinate locally and the total dosage of the drug in PLA-, PLGA-, and PCL-based coatings was 146, 750, and 1500 times lower, respectively, for the entire course of treatment. Note, that we encapsulated the maximum possible amount of the drug for our microchamber geometry. We suppose that changes in geometry with the increasing amount of the drug can enhance the therapeutic effect identified (Figure 5d).

**Figure 6:**
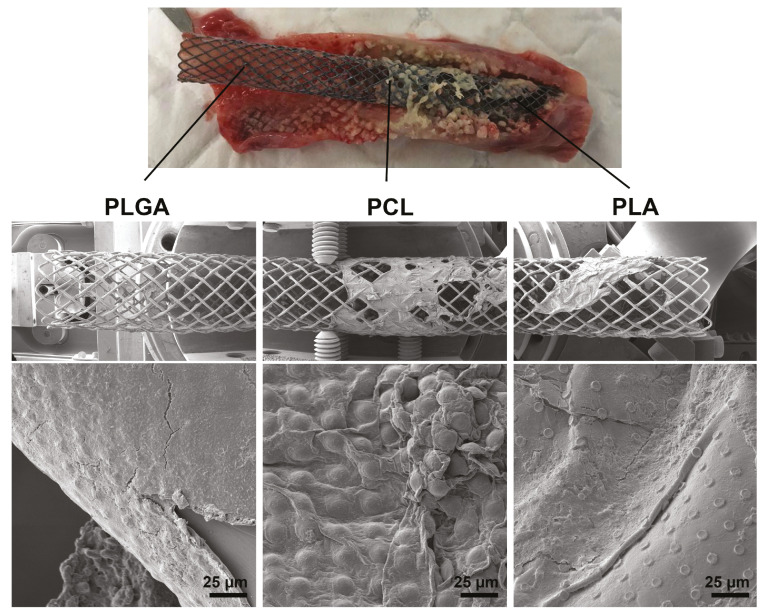
Biodegradation of PLGA, PCL, and PLA-based coatings 10 days after stent implantation in rabbit trachea. Photo of the trachea with a stent on top, SEM images of the coatings on the bottom.

SEM showed that PLGA-based films biodegraded almost completely 10 days after implantation into the rabbit trachea (Figure 6). The degradation of PLA-based film was less pronounced, although individual chambers were well visualized in the remaining areas of the film. The PCL-based film did not change its morphological state significantly. Long-term degradation of the PCL-based film on the stent can cause the aggravation of the inflammatory process—the reason for granulation tissue overgrowing by blocking the outflow of fluid from the tracheal mucosa [2]. Fluid accumulation is a favorable environment for bacterial colonization and biofilm formation, which is a typical problem with any type of implant [60].

While articles usually contain first screening studies only a few months after stent implantation [61], our research shows a significant development of granulation tissue already in the first 10 days in the control rabbit group. Therefore, we believe that this is the most critical period for anti-inflammatory therapy. In clinical practice, anti-inflammatory drugs are also usually injected in the first days after surgery into the trachea [51]. The most pronounced therapeutic effect from PLGA-based coating is possibly associated with slow drug’s release in the first few days, combined with rapid degradation and normalization of fluid outflow from the trachea mucosa.

A tracheal stent implantation is inevitably accompanied by the increasing secretion of mucus in the trachea and the own and pathogenic microflora development, always present in the airways. This reaction to stenting is a typical response to the presence of a “foreign body” in the trachea, which is reflected in many publications [60,62]. Microflora normalizes after stent epithelialization. We administered the antibiotic (gentamicin) daily by inhalation in all animal groups to avoid the pathological consequences associated with microbial contamination. Encapsulation and local delivery of antibacterial drugs is itself another promising area where the developed coatings can be applied in the future.

## 4. Conclusions

This study demonstrates that PLA-, PCL- and PLGA-based films with loaded microchamber arrays can be used as coatings for nitinol stents. Among these three types of microchamber, the PCL and PLA are seen as most resistant to mechanical damage when deposited on the delivery device. Thus, microchamber films made of these polymers make it possible to keep high-molecular-weight cargo in these microchambers. Low-molecular-weight therapeutic compounds such as methylprednisolone sodium succinate are practically not retained in PCL microchambers after immersion in deionized water (only 9.5% is left), while PLA-based microchambers retain methylprednisolone sodium succinate well (96.3%). PLGA, in comparison with PLA and PCL, is less stable for mechanical deformation, which leads to a significant loss of high-molecular-weight cargo when the medical device is delivered and placed. Low-molecular-weight methylprednisolone sodium succinate is washed away in significant part from the chambers at the synthesis stage, with 15.7% of the initial cargo remains while delivered. In vivo studies on rabbits have shown that both PLA and PLGA coatings have a significant therapeutic effect. A possible explanation for this phenomenon is that the residual amount of the drug in the PLGA film was sufficient to provide a therapeutic effect. The microchamber coating almost completely degrades after 10 days in the trachea. PLA-based microchambers took longer to degrade but contained the maximum amount of drug, which probably contributed to a more prolonged drug release into the tracheal mucosa. However, the therapeutic effect of PLGA coatings was slightly more pronounced; therefore, we consider the PLGA-based coating to be the most promising. The drug amount in the PCL-based microchambers was the smallest and insufficient to evaluate the therapeutic effect. In addition, long-term degradation of the PCL-based film on the stent can cause the aggravation of the inflammatory process (and the growth of granulation tissue) by blocking the outflow of fluid from the tracheal mucosa.

In summary, based on our studies, we propose that the approach to modify stents with widely used FDA-approved polymers in specific shape providing a sufficient loading of therapeutic cargo could be broadly applied for implantable medical devices as shown here on the particular example of the tracheal stent. Here, we discussed the advantages and drawbacks of microchamber film coatings made of pure polymers PLA, PLGA, and PCL. Further study could optimize polymer compositions considering the opportunity to blend the polymers to ensure proper therapeutic cargo encapsulation and mechanical properties. Overall, the method of fabrication and deposition of such microchamber film is robust and could be scaled up for manufacturing use.

## Data Availability

Data underlying the results presented in this paper are not publicly available at this time but may be obtained from the authors upon reasonable request.

## Supplementary Materials

The following are available online at https://www.mdpi.com/article/10.3390/pharmaceutics13091437/s1, Figure S1: The separation between microchamber array film and stent while deforming with the delivery system in case of the absence of an intermediate polymer layer between the film and nitinol; Figure S2: The L929 cell viability on films with PLA, PLGA, and PCL-based microchamber arrays 24 h after incubation; error bars correspond to standard deviation in 3 replications (a). CLSM image shows normal morphology of L929 cells 24 h after incubation on the PLA-based microchamber array surface. Green color corresponds to vital fluorescence dye calcein (b).
