# Supplementary material for: Patterned Drug-Eluting Coatings for Tracheal Stents Based on PLA, PLGA, and PCL for the Granulation Formation Reduction: In Vivo Studies
Source: Pharmaceutics. 2021 Sep 9;13(9):1437. doi: 10.3390/pharmaceutics13091437 (PMC8469052; doi:10.3390/pharmaceutics13091437)
Supplement: Supplementary file 1 [file pharmaceutics-13-01437-s001.zip › pharmaceutics-1333375-supplementary.pdf]

pharmaceutics

Supplementary Material: Patterned Drug-Eluting Coatings for Tracheal Stents Based on PLA, PLGA, and PCL for the Granulation Formation Reduction: In Vivo Studies

Olga A. Sindeeva, Ekaterina S. Prikhozhdenko, Igor Schurov, Nikolay Sedykh, Sergey Goryainov, Arfenya Karamyan, Ekaterina A. Mordovina, Olga A. Inozemtseva, Valeriya Kudryavtseva, Leonid E. Shchesnyak, Rimma A. Abramovich, Sergey Mikhajlov and Gleb B. Sukhorukov

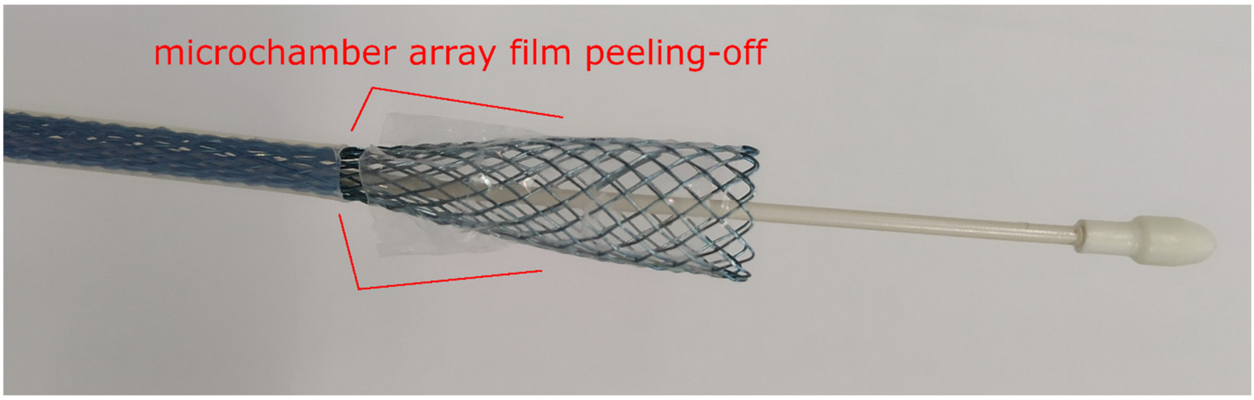

Figure S1. The separation between microchamber array film and stent while deforming with the delivery system in case of the absence of an intermediate polymer layer between the film and nitinol.

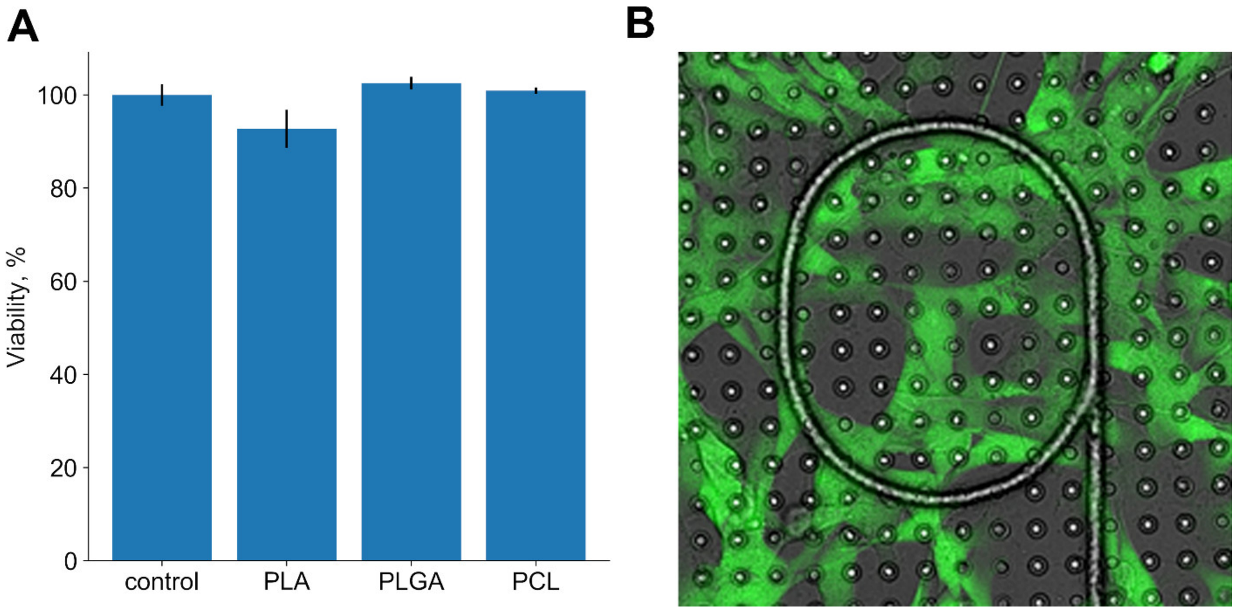

Figure S2. The L929 cell viability on films with PLA, PLGA, and PCL-based microchamber arrays 24 h after incubation; error bars correspond to standard deviation in 3 replications (**A**). CLSM image shows normal morphology of L929 cells 24 h after incubation on the PLA-based microchamber array surface. Green color corresponds to vital fluorescence dye calcein (**B**).
